# Simultaneous Patterning of Independent Metal/Metal Oxide Multi-Layer Films Using Two-Tone Photo-Acid Generating Compound Systems

**DOI:** 10.3390/nano2040312

**Published:** 2012-10-16

**Authors:** Christopher E. J. Cordonier, Hideo Honma

**Affiliations:** Materials & Surface Engineering Research Institute, Kanto Gakuin University, 1-1-1 Fukuura, Kanazawa, Yokohama 236-8501, Japan; Email: honma@kanto-gakuin.ac.jp

**Keywords:** photo-patterning, direct patterning, multi-layer films, multi-touch panel, iron, copper, lanthanides

## Abstract

(1) The photo-induced solubility and positive-tone direct photo-patterning of iron, copper and lanthanides chelated with 4-(2-nitrobenzyloxycarbonyl)catechol (NBOC) or 4-(6-nitroveratryloxycarbonyl)catechol (NVOC) was investigated. Photo-patterning of iron, copper, cerium, samarium, europium, terbium, dysprosium, holmium, erbium and lutetium complexes was accomplished. Continuous films were formed by the pyrolysis of metal complex films at 500 °C. (2) Based on the difference in the photo-reaction excitation wavelength profile of NBOC and NVOC complexes, a short and simple method for simultaneous micro-patterning of two independent films on each side of a transparent glass substrate was developed. Using the developed procedure, indium tin oxide and/or titanium oxide films were formed on each side of a quartz substrate without use of resist or etching.

## 1. Introduction

Development of display and interactive telecommunication technology increases the level of interaction between people and the virtual world, which increases the potential degree of communication. Advances in touch screen and display technology have significantly contributed to the development of the current state of telecommunication. For instance, the developments of capacitive type multi-touch panels and thin, high-resolution liquid crystal and organic electroluminescent displays have found extensive application in interactive visual communication products. In order to construct multi-touch panel displays for high-density devices, thin multilayer film structures are independently fabricated on both sides of optically transparent substrates for example borosilicate glass. Although there are many construction methods for multi-touch panels, we found a common design for capacitive type tactile sensors was transparent indium tin oxide (ITO) film complementary electrodes, which vary in pattern, on both sides of a glass or transparent polymer panel [1,2,3].

**Figure 1:**
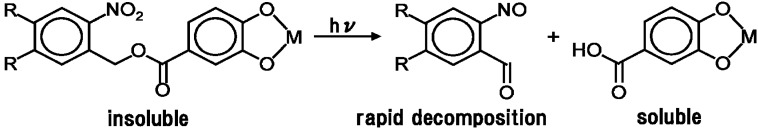
Photo-cleavage of nitrobenzyl ester derived complexes to produce carboxylic acid moiety bearing complexes [4].

We have reported a dual-function type of metal chelate complex that as a film can both be directly pattered and perform as a metal oxide precursor. This was accomplished with ortho nitrobenzyl esters of protocatechuic acid, 4-(2-nitrobenzyloxycarbonyl)catechol (NBOC; **1**) and 4-(6-nitroveratryloxycarbonyl)catechol (NVOC; **2**), metal complexes, where the corresponding protocatechuic acid complex was generated by the photo-induced cleavage of the ester (Figure 1) [4]. Contrast that can be used for patterning was achieved based on the selectively aqueous base soluble photo-generated carboxylic acid moiety bearing complex. The photoreaction has been confirmed by the extraction and characterization of the protocatechuic acid complex from irradiated films. In a following investigation, the photoreaction of free ligands and related derivatives has been monitored by NMR and the generation of the corresponding carboxylic acid was confirmed [5]. In that report, the relative dynamics of the photoreaction that occurs in the nitrobenzyl esters, related compounds and complexes under irradiation by a super high-pressure mercury lamp has been characterized.

Patterning of palladium, cobalt, nickel, titanium, niobium, tantalum, hafnium and indium tin has been (or will be) described elsewhere, where L/S 2–100 μm L-line, L-trench, bump and hole patterns and L/S 1.5 × 10 μm mesh patterns were prepared [4,5,6,7]. Controlling the pyrolysis and annealing conditions of indium tin complexes has also produced ITO films, which had relatively low electrical resistivity for a solution process. Here, photo patterning of iron, copper, and the lanthanides (except for promethium) with the **1** or **2** chelating ligands was investigated. Elaboration of metals that can be patterned using the **1** or **2** illustrates the versatility of this technique for potential application in situations where direct patterning from a wide selection of metals or metal mixtures may provide a solution to fabrication needs.

**Figure 2:**
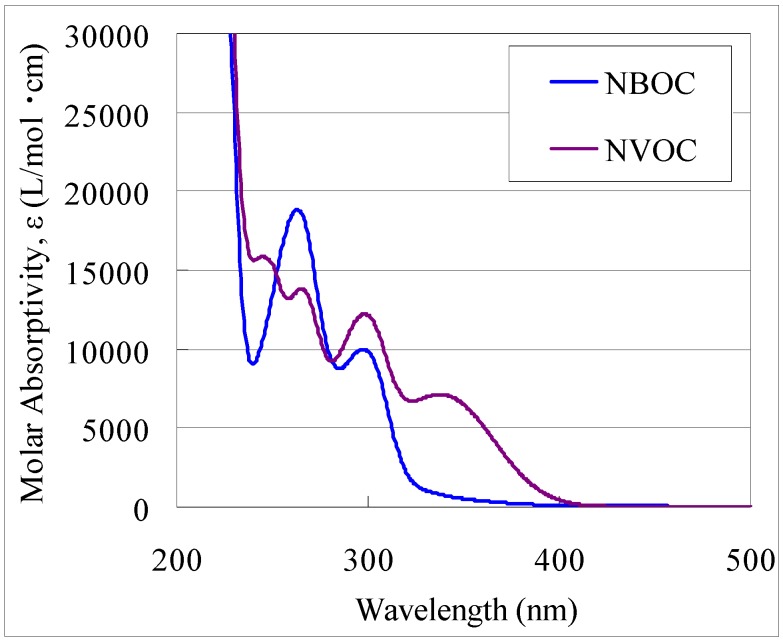
UV-Vis spectra the free ligands **1** (NBOC) and **2** (NVOC). Where NBOC is 4-(2-nitrobenzyloxycarbonyl)catechol, NVOC is 4-(6-nitroveratryloxycarbonyl)catechol.

Depending on the photo-reactive group, the photoreaction excitation wavelength profile varies. As described, compared to NBOC, the NVOC photoreaction wavelength profile extends to 400 nm from 350 nm (Figure 2). Reviews of photo-acid releasing groups that can be considered for this technique have been published [8,9,10,11,12]. Reaction of NVOC titanium complex under exposure through a short wavelength sharp cut filter (half intensity at 390 nm) has been shown to occur selectively, whereas the NBOC titanium complex didn’t significantly react under this condition [4]. Variation of the photoreaction excitation wavelength profile can be manipulated, introducing unique attributes that may be further utilized for the development of new devices and fabrication techniques. For instance, an additional dimension can be introduced to patterning by the introduction of color tone to the metal complex direct photo-patterning technique. In many instances, as in thin-film transistor displays and capacitive type multi-touch screens, multi-layered devices are composed of different patterned metal oxides that perform various functions. In these situations, the applied materials often differ in thickness and in etching characteristics and require individual construction taking care not to affect existing structures. For example, the etching rates and conditions of ITO and TiO_2_ are not similar although in the previously reported method [4], once exposed both precursors had been patterned under similar developing conditions, independent of film material and thickness. In this work, a simultaneous patterning process was developed for construction of independent metal oxide components on opposite surfaces (both sides) of a transparent substrate, independent of deposited film thickness or etching characteristics. This was accomplished through contriving a two-tone patterning technique; it was based on the finding that in the **1** or **2** metal complex patterning technique, developing characteristics are almost metal element independent. Almost all **1** or **2** metal complexes are (or can be engineered to be) developed in dilute TMAH within 30 s. Analysis of the pattern generation ability of the **1** or **2** iron, copper or lanthanide complexes in which the same developing conditions were used further exemplifies this aspect.

## 2. Results and Discussion

### 2.1. Photosolvolysis of Nitrobenzyloxycarbonylcatechol-Metal Solution Films

In addition to the metal elements reported so far, to explore the boundaries of the **1** (or **2**) metal complex direct patterning technique, the photo-induced solubility and patterning of **1** (or **2**) complex films of iron, copper and all the lanthanides (except for promethium) was examined. The photo-induced solubility dynamics of the **1** complex films of iron, copper and holmium as a representative of the lanthanides were investigated and the **2** complex film of holmium for comparison. The regions of the UV-Vis spectra defined in Table 1 were used to monitor the photo-induced solubility. 

**Table 1:** Experimental parameters and observations of nitrobenzyl 3,4-dihydroxybenzoate metal complex film photo-solvolysis.

Compound	Thickness Complex Film (nm)	Thickness Metal Oxide Film (nm)	*t*_e_ for Pattern (min)	Unexposed Film Area (TMAH 30 s)	Exposed Film Area (TMAH 30 s)	Monitored (nm)	Line Pattern Resolution (μm)	Trench Pattern Resolution (μm)
**1**-Fe	634 ± 1	50.5 ± 2.3	25	94%	0%	290–300	2	2 ^a^
**1**-Cu	574 ± 13	58.8 ± 5.5	30	85%	0%	280–310	2 ^b,c^	6 ^a,c,d^
**1**-La	–	–	20	100%	72%	250–320	–	–
**1**-Ce	541 ± 7	53.6 ± 1.2	30	100%	3%	250–320	2 ^e^	2
**1**-Pr	451 ± 8	49.9 ± 2.0	20	100%	19%	250–320	–	–
**1**-Nd	–	–	30	100%	100%	250–320	–	–
**1**-Sm	419 ± 4	50.2 ± 2.5	20	96%	1%	250–320	2 ^b,e^	2
**1**-Eu	479 ± 2	50.7 ± 2.0	30	100%	1%	250–320	2 ^e^	2
**1**-Gd	452 ± 6	56.6 ± 6.3	30	100%	49%	250–320	–	–
**1**-Tb	474 ± 4	50.3 ± 3.9	30	100%	1%	250–320	2 ^b^	2
**1**-Dy	373 ± 19	50.2 ± 4.6	20	99%	8%	250–320	2 ^b,e^	2 ^b^
**1**-Ho	457 ± 5	60.6 ± 2.4	15	100%	0%	250–320	2 ^b,e^	2
**2**-Ho	508 ± 7	59.5 ± 3.6	10	99%	1%	250–320		
**1**-Er	464 ± 2	51.6 ± 3.9	30	100%	0%	250–320	2	2
**1**-Tm	–	–	20	1%	0%	250–320	–	–
**1**-Yb	–	–	20	0%	0%	250–320	–	–
**1**-Lu	428 ± 5	46.5 ± 1.6	20	99%	0%	250–320	2 ^d^	2

^a^: Excessive film material removal, excessively wide trench/excessively narrow line; ^b^: Deficient film material removal, excessively narrow trench/excessively wide line; ^c^: Residual material in irradiated areas; ^d^: Delamination of patterned film; ^e^: Inter-pattern residual film.

**Figure 3:**
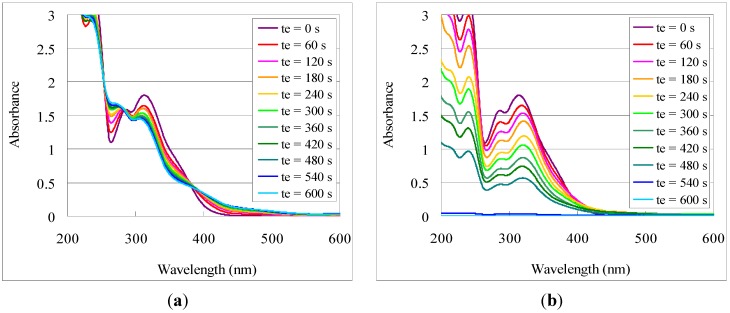
Photo-induced solubility of **2**-Ho films, monitored by UV-Vis spectra of **2**-Ho films (**a**) exposed to UVB irradiation for time ***t***_e_; and (**b**) after developing the irradiated films.

**Figure 4:**
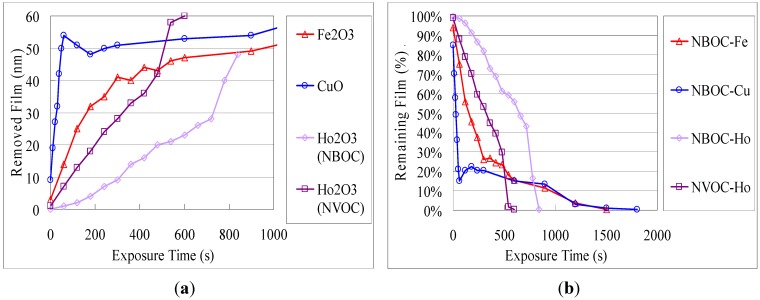
Photo-solvolysis of nitrobenzyl 3,4-dihydroxybenzoate metal complexes in terms of (**a**) the remaining fraction of the total complex film; and (**b**) the amount of removed film in terms of metal oxide film.

Figure 3 shows the overlaid UV-Vis spectra of **2**-Ho films with UV exposure before and after developing as a representative example. In this case, change of the UV-Vis absorption profile with irradiation time (***t***_e_) was observed, reflecting the progression of the photochemical reaction. Upon developing, the absorption intensity decreased proportional to ***t***_e_, leaving a profile similar to the unexposed film. This behavior coincides with the previously observed results for titanium and indium tin, suggesting a similar photo-reaction [4,5]. Deduced in a similar manner, the photo-induced solubility dynamics of **1**-Fe, **1**-Cu, **1**-Ho, and **2**-Ho are shown in Figure 4 in terms of removed metal oxide film and percentage of remaining film after exposure and developing. Similar to the trend observed for the difference in photo-efficiency between titanium and indium tin, photo-efficiency of **1**-Cu was highest followed by **1**-Fe then **1**-Ho. Similarly, the holmium complex ligand-metal charge transfer (LMCT) absorption overlap of the NBOC excitation was strongest followed by that of the iron complex and least for that of the copper complex. The LMCT of the iron and copper complexes were relatively broadened into the visible spectrum compared to that of holmium. The photo-efficiency of **2**-Ho was significantly higher than that of **1**-Ho. This finding may be explained as being due to the extended photoreaction profile of the NVOC system, which extends beyond the LMCT absorption, increasing the amount of effective irradiation on the NVOC moiety and therefore increasing the total system quantum yield. Although the initial photo-removal rate was faster for copper and iron, after 85% of the **1**-Cu and 75% of the **1**-Fe was removed the removal rate abruptly decreased. The reason for this phenomenon is unclear but it may be due to the influence of an intermediate species or a counteractive photo-induced polymerization or hydrolysis reaction.

**Figure 5:**
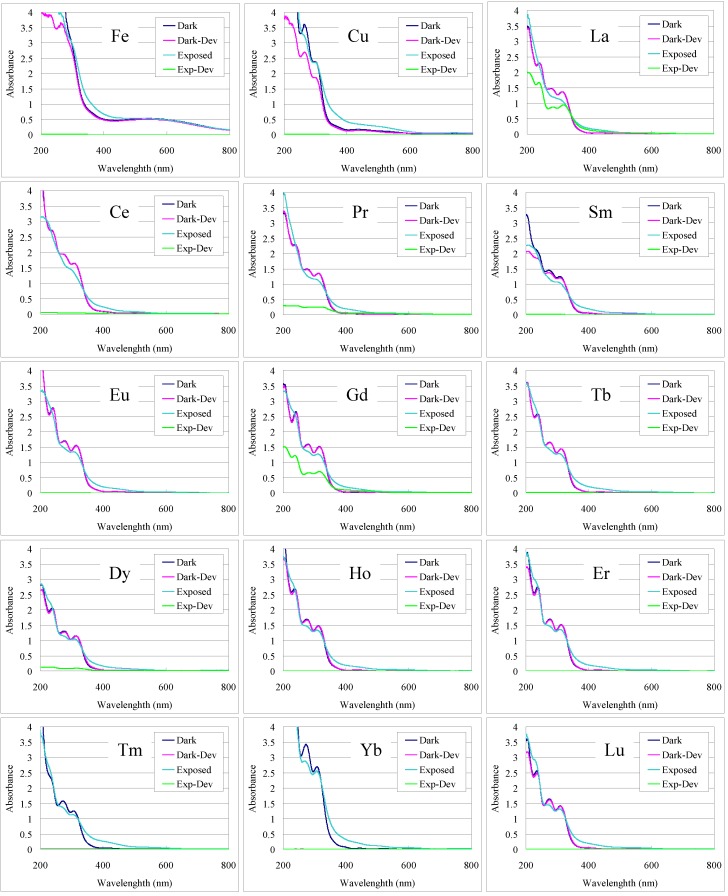
UV-Vis spectra of the masked (Dark) and exposed (Exposed) areas of **1** + metal films before and after developing in 0.25 wt % TMAH (Dev).

**Figure 6:**
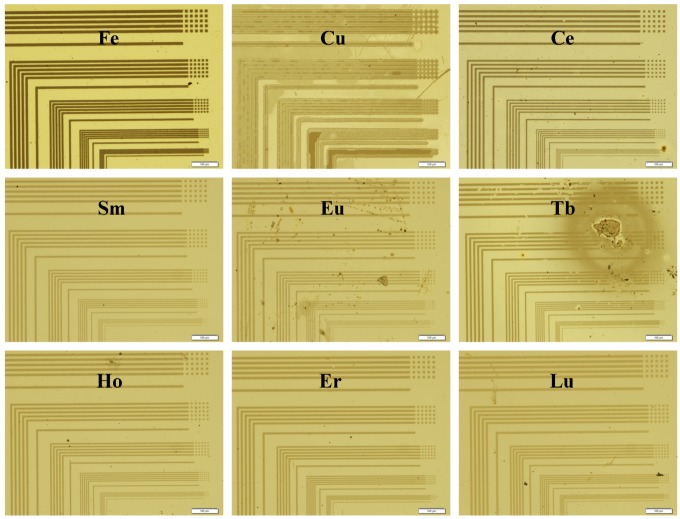
Microscope images of the 2, 4, 6, 8 and 10 μm trench and hole patterned **1** + metal films on Pyrex substrates after pyrolysis at 500 °C.

Next, the photo-patterning ability of the iron, copper and lanthanide complexes of **1** and the **2**-Ho complex was evaluated with the L-line photomask, using the known procedure [4,5]. The ***t***_e_ used for patterning, as listed in Table 1, was determined as the minimum ***t***_e_ to completely remove a film for the four measured films. To evaluate the lanthanides other than holmium and promethium, typically 20 min. was used and 30 min. in some cases when dissolution was incomplete. The overlaid UV-Vis spectra of the film before and after exposure and developing in Figure 5 depict the contrast or potential for pattern formation. Microscope images of the L-trench and hole patterns of selected annealed films (Figure 6) illustrate the patterning ability of the complex films and the continuity of the resulting pyrolyzed films.

The numerical values for the remaining films of the exposed and unexposed regions after developing listed in Table 1 exhibit the contrast attained for each film. Contrast of the solubility in developer between the exposed and unexposed areas is required for patterning. Of the lanthanides, cerium, samarium, europium, terbium, dysprosium, holmium, erbium and lutetium could be patterned as a complex of **1**. Some contrast was observed in lanthanum, praseodymium, and gadolinium films, although the photo-efficiency was comparatively lower and no contrast was observed in neodymium, thulium and ytterbium. In the case of neodymium, neither the exposed or unexposed areas of the film were soluble in developer. While for thulium and ytterbium, both the exposed and unexposed areas of the film were completely soluble. In the cases contrast was not sufficiently attained, the film thickness could not be measured by surface profile. Insufficient contrast under the conditions examined may be improved by use of alternate metal reagents, solvents or additives.

### 2.2. Two-Layer Film Patterning

A conventional method for depositing transparent electrodes on both sides of a transparent substrate may be long and energy consuming. For example: (i) Sputter ITO onto side-1 of the substrate; (ii) sputter ITO onto side-2 of the substrate; (iii) coat side-1 of the substrate with photoresist; (iv) expose with pattern-1; (v) develop, (vi) hard bake; (vii) coat side-2 of the substrate with photoresist; (viii) expose with pattern-2; (ix) develop; (x) hard bake; (xi) etch ITO; (xii) strip resist and remove scum (ashing). In this example, the films on both sides of the substrate consist of the same material and are of the same thickness. But in instances where the material on both sides of the substrate are not similar in etching characteristics or do not bear the same etching requirements, each side must also be etched independently, further lengthening and complicating the fabrication procedure. In situations where etching rates or film thickness vary, prolonged exposure of one side may result in undercut.

**Figure 7:**
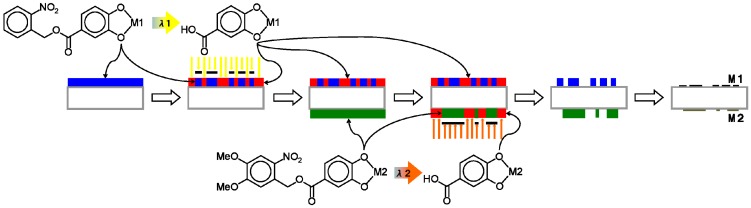
Procedure (Method A) for simultaneous photo patterning of independent metal/metal oxide bi-layer films using two-tone photoacid generating nitrobenzyl ester derived complexes.

**Figure 8:**
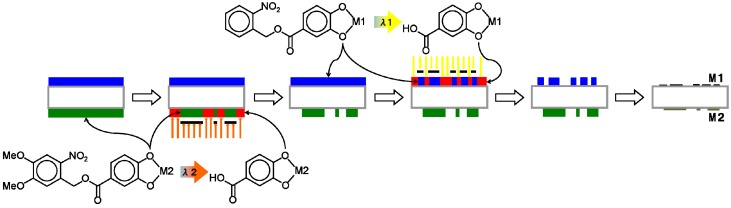
Procedure (Method B) for simultaneous photo patterning of independent metal/metal oxide bi-layer films using two-tone photoacid generating nitrobenzyl ester derived complexes.

As shown in Section 2.1. and elsewhere [4,5,6,7], many metal complexes can be patterned by developing under the same conditions in diluted TMAH for 30 s, completely independent of the element and film thickness. Similarly, the various patterned metal oxide films have been formed by pyrolysis under the same conditions. Based on the material independence of the **1** or **2** metal complex patterning process and on the photoreaction selectivity between **1** and **2** with 370–400 nm irradiation (Figure 2), two methods for simultaneously patterning two independent metal oxide films on a transparent substrate were devised. One method (Method A) is as illustrated in Figure 7: (i) Coat side-1 of the substrate with the NBOC metal-1 complex; (ii) expose side-1 to irradiation between 200 and 370 nm with pattern-1; (iii) coat side-2 of the substrate with the NVOC metal-2 complex; (iv) expose side-2 to irradiation between 370 and 400 nm with pattern-2; (v) submerse in developer; (vi) oxidize to give patern-1 of metal oxide-1 on side-1 and patern-2 of metal oxide-2 on side-2. The other method (Method B) is as illustrated in Figure 8: (i) Coat side-1 of the substrate with the NBOC metal-1 complex; (ii) coat side-2 of the substrate with the NVOC metal-2 complex; (iii) expose side-2 to irradiation between 370 and 400 nm with pattern-2; (iv) develop side-2; (v) expose side-1 to irradiation between 200 and 370 nm with pattern-1; (vi) develop side-1; (vii) oxidize to give patern-1 of metal oxide-1 on side-1 and patern-2 of metal oxide-2 on side-2. Each method has tradeoffs. Method A only requires one developing step although each complex coat must be dried individually; in Method B, both coatings can be dried simultaneously. Depending on production equipment and conditions one method may sanction advantages over the other.

In the event M1 and M2 differ, there are several factors to consider as to which element to use as M1 and which to use as M2. Two important factors are the photo-induced solubility selectivity and necessary exposure time. As illustrated, the complex LMCT absorption affects the photoreaction efficiency and use of the cut-filter significantly reduces the amount of incident reaction inducing irradiation. In order to maximize photo-selectivity and minimize exposure time, the less reactive element should be applied as M1 and the faster reacting element as M2. For the example of TiO_2_ on one side and ITO on the other of quartz glass, more slowly reacting titanium was used as M1 and faster reacting indium tin as M2. With this selection the total exposure time, ***t***_e1_ + ***t***_e2_ = 25 min. If indium tin were used as M1 and titanium as M2, the total exposure time (***t***_e1_ + ***t***_e2_) would require 62 min. and prolonged exposure of the M2 film may increase the probability of influence to the M1 film.

**Figure 9:**
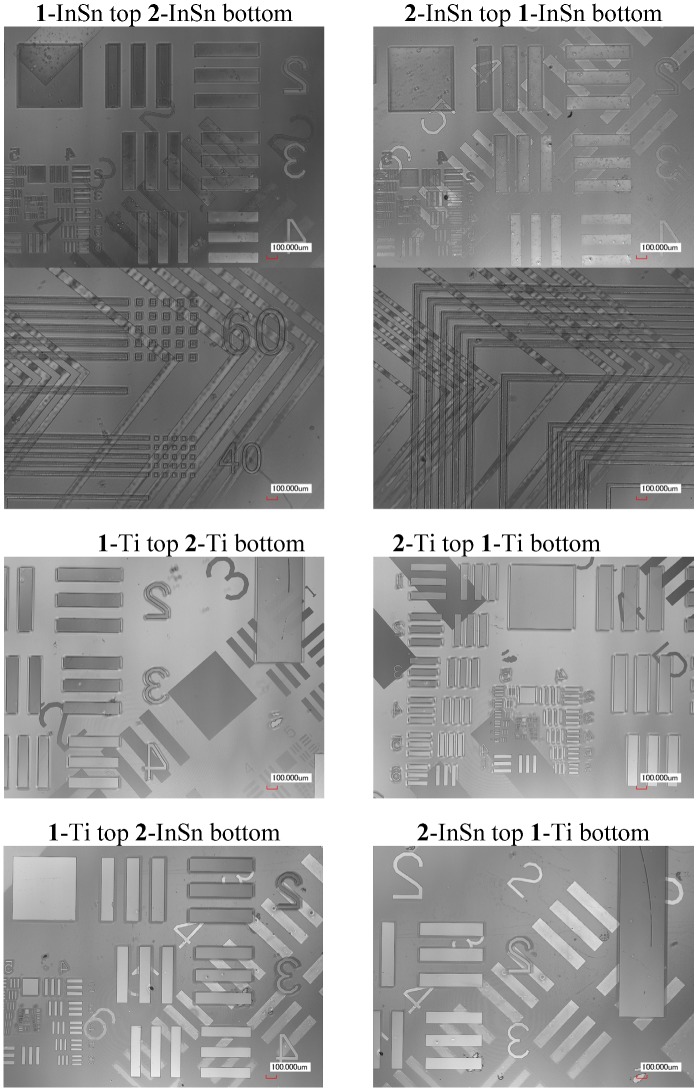
Laser microscope images of quartz substrates with patterned metal oxide films on both sides deposited by two-tone patterning Method A.

**Figure 10:**
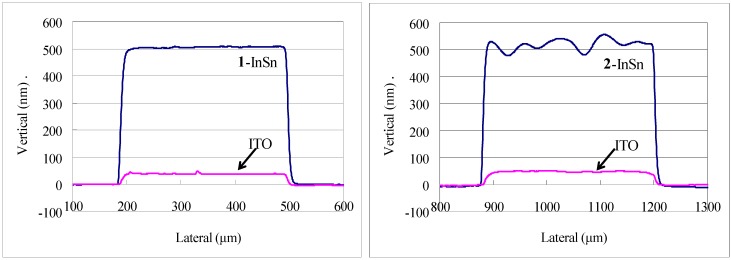
Surface profiles of patterned **1**-InSn and **2**-InSn films on each side of a quartz substrate deposited by two-tone patterning Method A and the resulting indium tin oxide (ITO) films formed by pyrolysis at 500 °C.

The dual-side patterned films in Table 2 were produced by Method A and B. Selected laser microscope images of samples produced using Method A are displayed in Figure 9. Figure 10 shows the profile of a 0.3 mm line of **1**-InSn and **2**-InSn films on each side of a quartz substrate before and after pyrolysis at 500 °C. The final results of either method for production of films in Table 2 were found to be equivalent. In Method A, after side-1 was exposed, side-2 was coated and dried at 100–120 °C. Despite the exposure to elevated temperature, the irradiated material on side-1 was completely removed upon developing in all cases examined. This exhibits the thermally inert nature of the photoproduct and compatibility of these compounds to this method. The electrical and optical properties and analysis of the respective ITO or TiO_2_ films formed by pyrolysis of the InSn and Ti complexes under these conditions have been reported [4].

**Table 2:** Dual-sided films.

Sample Side-1/Side-2	Compound Side-1/Side-2	Photomask	Dry Film-1 (nm)	Pyrolyzed Film-1 (nm)	Dry Film-2 (nm)	Pyrolyzed Film-2 (nm)
ITO/ITO	**1**-InSn/**2**-InSn	1951 USAF	506 ± 2	39.2 ± 1.6	518 ± 20	48.4 ± 1.5
ITO/ITO	**1**-InSn/**2**-InSn	L-line/trench	592 ± 16	49.9 ± 1.2	714 ± 68	67.5 ± 1.4
TiO_2_/TiO_2_	**1**-Ti/**2**-Ti	1951 USAF	324 ± 4	24.9 ± 2.8	400 ± 8	38.1 ± 3.5
TiO_2_/ITO	**1**-Ti/**2**-InSn	1951 USAF	334 ± 3	24.1 ± 2.3	617 ± 20	53.2 ± 2.1

### 2.3. Pyrolysis of Patterned Complex Films

**Figure 11:**
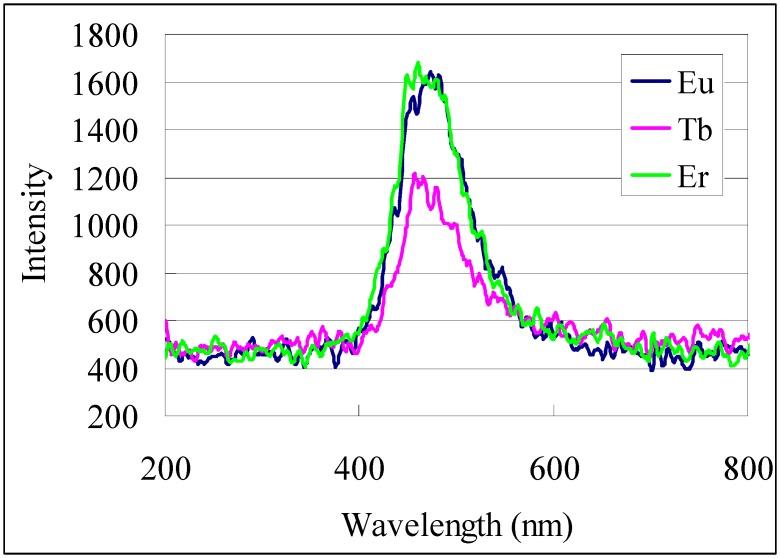
Cathode luminescence spectra of patterned metal oxides formed from pyrolysis of **1**-Eu, **1**-Tb and **1**-Er films at 500 °C.

The transformation of organometallic precursors to various materials can be accomplished by many methods, but in this investigation we only performed a simple thermal treatment of the complex films at 500 °C in air. Metal oxide films formed by pyrolysis of the respective complex films showed reasonable optical characteristics compared with known materials. Except for copper oxide, the XRD patterns from the metal oxides formed here were to feint for characterization. The copper oxide films however showed appropriate diffraction patterns for the CuO monoclinic crystal phase. The cathode luminescence of the patterned europium oxide, terbium oxide, and erbium oxide films selected as representatives of the lanthanide oxide films was investigated. All three oxide films had an emission peak centered at about 470 nm as shown in the cathode luminescence spectra (Figure 11).

Formation of the appropriate metal oxide from the respective metal complex was confirmed by EPMA. Table 3 lists the elemental analysis of the patterned metal oxide films. The precursor complexes are composed of carbon, nitrogen, oxygen, and the chelated metal. Theoretically, the pyrolyzed films could contain any of these elements and the glass substrate could contain silicon. For each sample, the oxide, nitride, carbide, and silicide formation affinity and the glass infusion rate may vary depending on the metal. In addition, carburization may leave carbon residue in or on the film, carbon monoxide or carbon dioxide may be absorbed from the atmosphere, and carbon based fouling may be transferred to the sample during handling. Nitrogen was not detected in any of the samples, and as shown in Table 3, the carbon content varied among the samples. Given the detection of carbon on the bare substrate, carbon presence in other samples may have been transferred during handling. The respective film metals, silicon from the glass and oxygen from both the pyrolyzed films and the glass were appropriately detected. In the case of ITO formation, an appropriate 5–10 atom % tin content with respect to indium and tin was observed in all three cases.

**Table 3:** EPMA elemental analysis of films formed by pyrolysis of metal complexes at 500 °C in an air atmosphere.

Compound	Metal	[Metal] (atom %)	[O] (atom %)	[C] (atom %)	[Si] (atom %)	Sample Current (nA)
**1**-Fe	Fe	4.0	28.7	2.4	64.9	26.3
**1**-Cu	Cu	3.0	24.3	N.D. *	72.2	26.5
**1**-Ce	Ce	2.5	23.3	N.D.	74.2	25.2
**1**-Sm	Sm	1.1	45.8	1.8	51.3	16.5
**1**-Eu	Eu	2.0	33.0	N.D.	65.0	27.6
**1**-Tb	Tb	3.2	23.4	4.0	69.4	24.4
**1**-Dy	Dy	1.3	44.2	N.D.	54.5	16.6
**1**-Ho	Ho	3.7	16.4	4.1	75.8	13.9
**1**-Er	Er	2.5	28.5	N.D.	69.0	14.1
**1**-Lu	Lu	2.3	16.6	32.4	48.8	14.9
**1**-InSn/	In + Sn	1.1 + 0.1	28.8	3.9	66.1	16.0
**2**-InSn	In + Sn	1.5 + 0.1	29.8	1.7	66.9	16.4
**1**-Ti/	Ti	1.2	35.0	4.8	59.0	16.0
**2**-InSn	In + Sn	1.0 + 0.1	36.1	4.0	58.8	15.4
**1**-Ti/	Ti	1.0	42.0	2.5	54.5	16.2
**2**-Ti	Ti	1.1	40.9	1.4	56.6	16.7
Substrate	–	–	28.1	6.1	65.8	15.6

* N.D. denotes not detected.

Depending on the complex, coating solution solvent, drying conditions, heating rate, pyrolysis temperature and gas atmosphere during pyrolysis among other parameters, the film nano-structure, crystal structure, porosity, elemental composition/stoichiometry (carbon/oxygen/nitrogen/sulfur/hydrogen ratio to metal), uniformity, continuity, physical and chemical properties of the resulting inorganic material may be controlled. More detailed investigation of the complexes to inorganic material transformations, analysis of the formed materials and transformation condition-material property correlations will be presented elsewhere. Control of the film properties by the solution and transformation parameters may enable tailoring the formed material to specific requirements.

## 3. Experimental Section

2-Nitrobenzyl 3,4-dihydroxybenzoate (**1**), and 6-nitroveratryl 3,4-dihydroxybenzoate (**2**) were prepared as previously reported [4]. Other reagents were used as purchased. Fused quartz glass (50 × 50 × 1t mm) was cleaned by reactive (oxygen) ion etching with a Samco RIE-10R prior to coating using a Mikasa 1H-D7 spin coater. The light source used for the photoreactions was parallel beam irradiation from an Ushio 250W super-high pressure mercury-xenon lamp USH-250BY through a 75 mm collimator; 0.626 mW at 245 nm, 15 mW at 310 nm and 67 mW at 365 nm. For irradiation of the metal complex of **2** on the second side in the simultaneous deposition of patterned films on both sides of a transparent substrate, when a sharp cutoff filter (Sigma SCF-50S-39 L, 50% T at 390 nm) was in place between the light source and photomask; 0.019 mW at 245 nm, 0.021 mW at 310 nm, 0.630 mW at 365 nm. UV-Vis spectroscopy was performed on a Hitachi U-3310 spectrometer. Microscopy was performed with a Keyence VK-9710 laser scanning microscope or with an Olympus BX51 optical microscope. Film thickness and profile were measured on a Veeco Dektak 150 stylus profiler. X-ray diffraction (XRD) patterns were measured on a Bruker D8 Discover system by detector scan (ω = 0.2°) using monochromated Cu Kα radiation. Cathode luminescence analysis of selected metal oxide films was performed on a Shimadzu SPG-120S spectrometer (Figure 11). Elemental analysis of the pyrolyzed metal complex films was performed with a Shimadzu EPMA-1610 electron probe microanalyzer (EPMA) using an acceleration voltage of 15.0 kV, beam size of 100 μm, and sample current listed in Table 3. Samples were sputtered with gold prior to EPMA.

### 3.1. Preparation of Photo-Reactive Solutions

The photo-reactive metal solutions were prepared by dissolving ligand, **1** or **2**, (2.50 mmol), 2-methoxyethoxyacetic acid (336 mg, 2.50 mmol), and the metal source reagent (2.50 mmol) in solvent to give a final volume of 5.0 mL. The solvents used were 2-methoxyethanol or 4:1:1 ethyl acetate/γ-butyrolactone (GBL)/*N*,*N'*-dimethylacetamide (DMA). The mixtures were agitated ultrasonically in a water bath at 60 °C until dissolution was complete. Solution compositions evaluated are shown in Table 4. All solutions were filtered through a PTFE membrane filter prior to use. The solutions of **1** or **2** chelated indium tin (0.5 M) or titanium (0.25 M) were prepared as reported [4].

**Table 4:** Photo-reactive metal complex coating solution composition.

Ligand	Metal Reagent	Amount for 2.50 mmol of Metal (mg)	Solvent
**1**	Fe(AcAc)_3_	882	2-Methoxyethanol
**1**	Cu(OAc)_2_	454	2-Methoxyethanol
**1**	La(AcAc)_3_·nH_2_O	1158	Ethyl lactate/GBL/DMA
**1**	Ce(AcAc)_3_·nH_2_O	1208	Ethyl lactate/GBL/DMA
**1**	Pr(AcAc)_3_·nH_2_O	1219	Ethyl lactate/GBL/DMA
**1**	Nd(AcAc)_3_·nH_2_O	1152	Ethyl lactate/GBL/DMA
**1**	Sm(AcAc)_3_·nH_2_O	1249	Ethyl lactate/GBL/DMA
**1**	Eu(AcAc)_3_·nH_2_O	1213	Ethyl lactate/GBL/DMA
**1**	Gd(AcAc)_3_·nH_2_O	1181	Ethyl lactate/GBL/DMA
**1**	Tb(AcAc)_3_·nH_2_O	1316	Ethyl lactate/GBL/DMA
**1**	Dy(OAc)_3_·nH_2_O	1025	Ethyl lactate/GBL/DMA
**1**	Ho(OAc)_3_·nH_2_O	1031	Ethyl lactate/GBL/DMA
**2**	Ho(OAc)_3_·nH_2_O	1031	Ethyl lactate/GBL/DMA
**1**	Er(AcAc)_3_·nH_2_O	1287	Ethyl lactate/GBL/DMA
**1**	Tm(OAc)_3_·nH_2_O	1077	Ethyl lactate/GBL/DMA
**1**	Yb(OAc)_3_·nH_2_O	1056	2-Methoxyethanol
**1**	Lu(AcAc)_3_·nH_2_O	1302	Ethyl lactate/GBL/DMA

### 3.2. Determination of Photo-Etching Rate

The respective solutions (0.5 mL) were spin-coated onto quartz plates (50 × 50 × 1t mm) at 1000 RPM then dried at 100–120 °C for one hour on a hotplate. The resulting films were then exposed to irradiation from the Hg-Xe lamp for time ***t***_e_ before developing by submersion in aqueous 0.25 wt % tetramethylammonium hydroxide (TMAH) solution for 30 s. The thickness of each of the unexposed films was measured from the surface profile between the base plate and the film surface. The listed values are the average of three measurements. The amount of film etched (Figure 4a) was determined by the difference in the UV-Vis absorption spectra before and after etching, monitoring the area specified in Table 1 standardized in terms of resulting metal oxide film attained upon pyrolysis at 500 °C.

### 3.3. Patterned Film Formation Procedure

Single film patterning was performed according to the reported procedure [4]. The complex film thickness of films patterned through the custom L-line photomask with 40 × 10 mm area was measured by stylus profiler by the height difference between the substrate base and the remaining film in the 40 × 10 mm unexposed area. The UV-Vis spectrum of the patterned samples was also measured using the dark 40 × 10 mm area and the exposed area next to it. The patterned films were heated to 500 °C at a rate of 10 °C/min. then baked at 500 °C in air for 1 h in an electric furnace to produce the corresponding patterned metal oxide film, for which film thickness was measured by stylus profiler. The removed film thickness values were calculated from the UV-Vis spectra standardized with the correlation between metal complex film absorption and the corresponding metal oxide film thickness of this area.

Simultaneous deposition of patterned films on both sides of a transparent substrate was performed as follows. Method A (Figure 7): The respective **1**-metal solution (0.5 mL) was spin-coated onto one side (side-1) of a quartz plate (50 × 50 × 1t mm) at 1000 RPM then dried at 100–120 °C for 1 h on a hotplate. The resulting film on side-1 was then exposed to irradiation from the Hg-Xe lamp through a photomask in position-1 for time ***t***_e1_. Next, the respective **2**-metal solution was similarly coated onto the other side (side-2) of the quartz plate, dried then exposed to irradiation from the Hg-Xe lamp through a photomask in position-2 for time ***t***_e2_ through the sharp cutoff filter before developing by submersion in aqueous 0.25 wt % TMAH solution for 30 s. The patterning parameters applied are as specified in Table 5. Finally, the patterned films were heated to 500 °C at a rate of 10 °C/min. then baked at 500 °C in air for 1 h in an electric furnace. The resulting metal oxide films were observed by XRD and patterns confirmed by laser or optical microscopy and profilometery from which selected images and profiles are shown in Figure 9 and Figure 10. The photomasks used were either a custom L-line mask or a positive 1951 USAF test pattern. The photomask position-1 and position-2 were aligned 45° from each other in such a way the patterns on each side would overlap.

Method B (Figure 8): The respective **1**-metal solution was coated onto one side (side-1) of the quartz plate and **2**-metal solution onto the other side (side-2) then dried. Next, side-2 was exposed to irradiation from the Hg-Xe lamp through the sharp cutoff filter and a photomask in position-2 for time ***t***_e__2_, developed, then side-1 was exposed to irradiation from the Hg-Xe lamp through a photomask in position-1 for time ***t***_e__1_ then developed. Finally, the patterned films were pyrolyzed at 500 °C to give the metal oxide films.

**Table 5:** Parameters for two-tone patterning.

Compound	Light Source	*t*_e_ for Pattern (min)
**1**-InSn	Hg-Xe	2
**1**-InSn	Hg-Xe + Cut-filter	>30
**2**-InSn	Hg-Xe	2
**2**-InSn	Hg-Xe + Cut-filter	15
**1**-Ti	Hg-Xe	10
**1**-Ti	Hg-Xe + Cut-filter	>120
**2**-Ti	Hg-Xe	10
**2**-Ti	Hg-Xe + Cut-filter	60

## 4. Conclusions

Direct patterning of the iron, copper and lanthanide complex of **1** or **2** films was investigated. Among these, iron, copper, cerium, samarium, europium, terbium, dysprosium, holmium, erbium, lutetium, lanthanum, praseodymium and gadolinium films were those found to be positive-tone patterned under similar conditions as reported for titanium and indium tin [4]. Almost all the metals could be patterned; however, neodymium, thulium and ytterbium showed no contrast under these conditions. Despite many known applications for the examined metals, only the patterning dynamics and the ability to form a pattern on preserved oxide films from the respective complex were investigated. These metal oxide films have been known as components of dielectric, optical, photoluminescent, photovoltaic, superconducting and magnetic film materials [13,14,15,16]. Potential applications include gate material in MOSFETs and dielectric films for capacitors, among a variety of functional materials for electronics, sensors and other devices. This survey contributed to the library of materials that can be used for patterned film construction with this photo-patterning technique.

In addition to expansion of the applicable patternable metal library, efficient methods for simultaneous patterning of two films of same or varying material on opposite sides of a transparent substrate were developed based on the varying excitation profiles of NBOC and NVOC moieties. As an example, transparent electrodes were simultaneously formed on both sides of a quartz substrate, simulating capacitive type multi-touch panel construction. This two-tone patterning technique is an invariant to the pattern dimensions and final film material etching characteristics on each side of the substrate, making it adaptable to construction of many dual-sided multi material structures. In other accounts, which are to be published in the near future, pyrolysis techniques that generate lower resistance ITO from the indium tin complexes and selective electroless plating on specialized metal oxide structures that can be patterned in the same manner as described here have been established. Application of the selective metallization technique to the simultaneous dual-side patterning technique adds another dimension to creative possibilities. In addition to transparent tactile sensors, this malleable technique may find use for simultaneous deposition of functional films on both sides of transparent substrates that may be applicable to the construction of MEMS, NEMS and other optical devices.
